# Supplementary figures and images for: A deep learning algorithm for 3D cell detection in whole mouse brain image datasets
Source: PLoS Comput Biol. 2021 May 28;17(5):e1009074. doi: 10.1371/journal.pcbi.1009074 (PMC8191998; doi:10.1371/journal.pcbi.1009074)

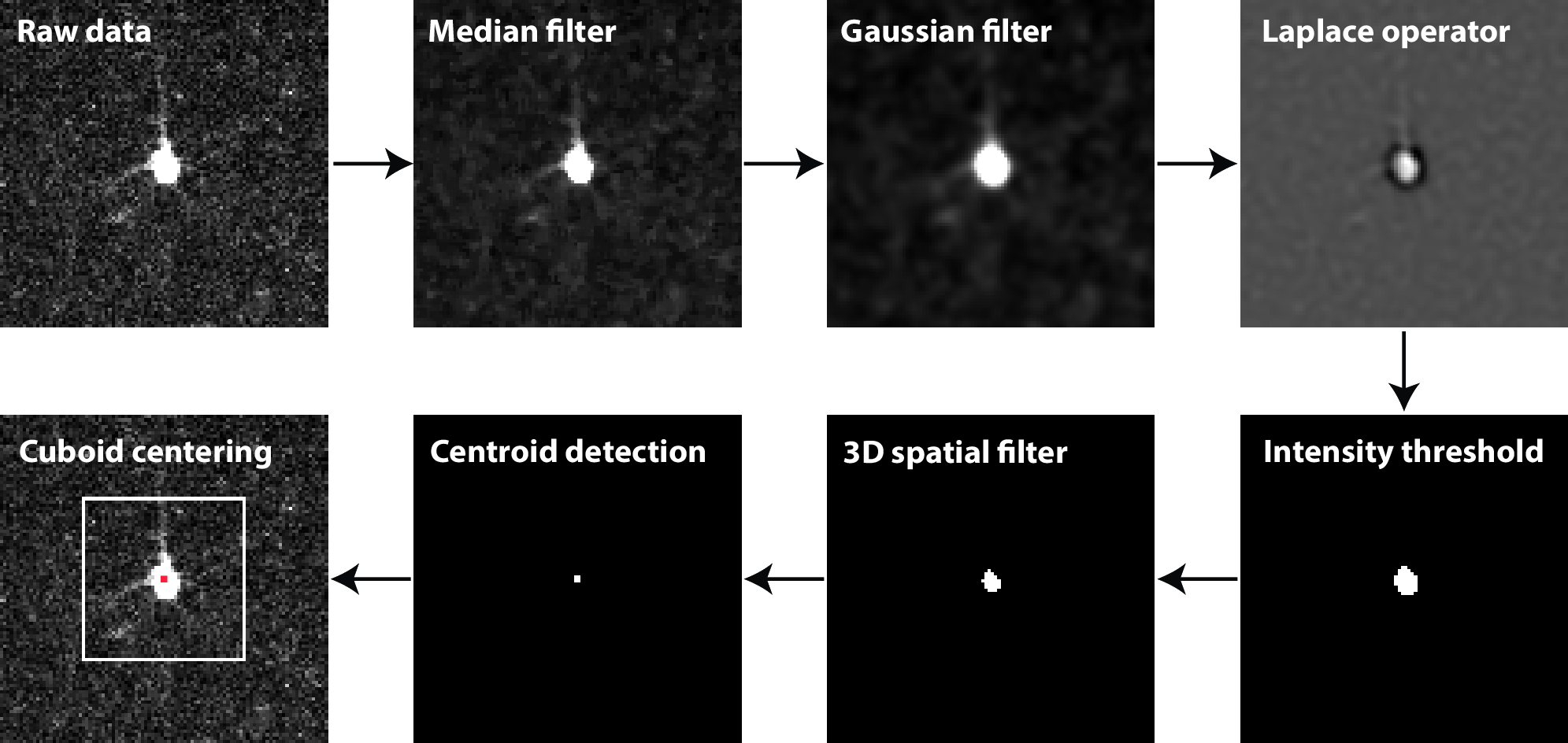

Supplement: S1 Fig — Upper row: from left to right, the raw image is median filtered to remove noise. A Laplacian of Gaussian is then performed to enhance small, bright structures such as the cell soma. Lower row: from right to left, the image is thresholded and a 3D ellipsoidal filter is used to remove small, non-cellular objects (not shown in this image plane). The centroid of the resulting object is then used to center the cuboid of data that it passed to the deep learning classification network. Images shown are 100 μm x 100 μm, and the cuboid is 50 μm x 50 μm (and 100 μm in the third dimension). (TIF) [file pcbi.1009074.s001.tif]

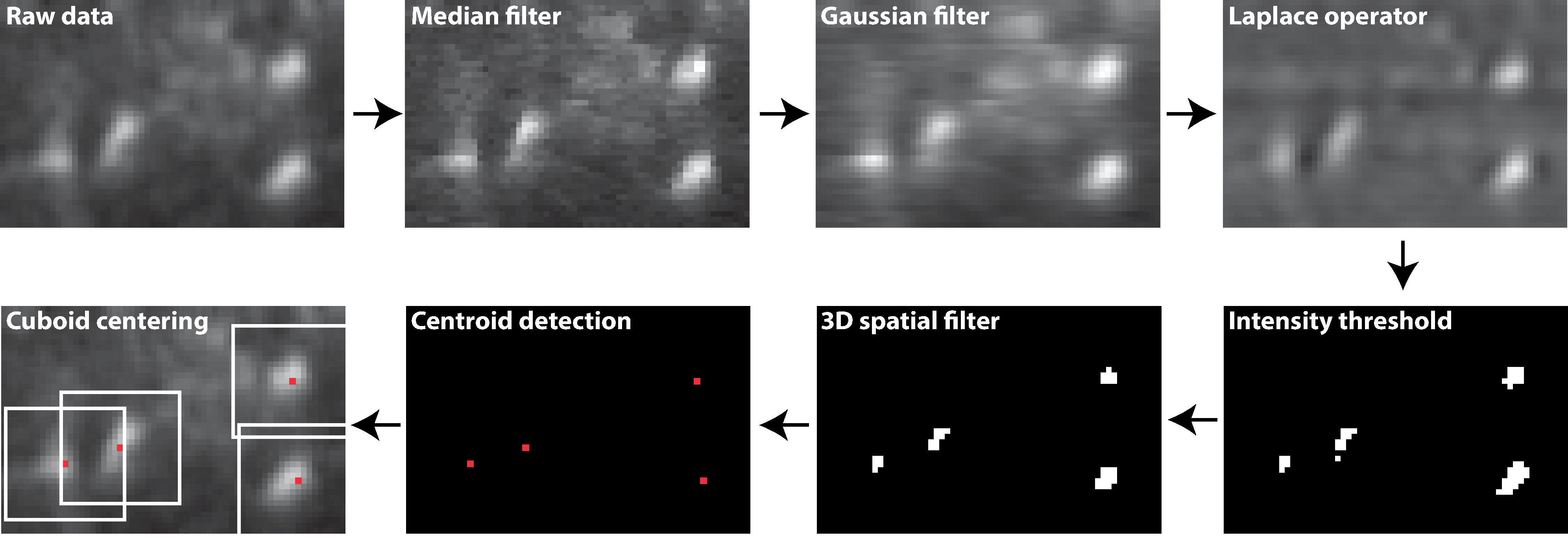

Supplement: S2 Fig — Upper row: from left to right, median and Laplacian of Gaussian filtering, as in S1 Fig. Lower row: from right to left, thresholding, filtering and centroid calculation, as in S1 Fig. Unlike 2D analysis, individual objects (cells) are correctly distinguished, and not merged, or erroneously split. Images shown are 205 μm x 143 μm, and the cuboid is 50 μm x 100 μm (and 50 μm in the third dimension). (TIF) [file pcbi.1009074.s002.tif]

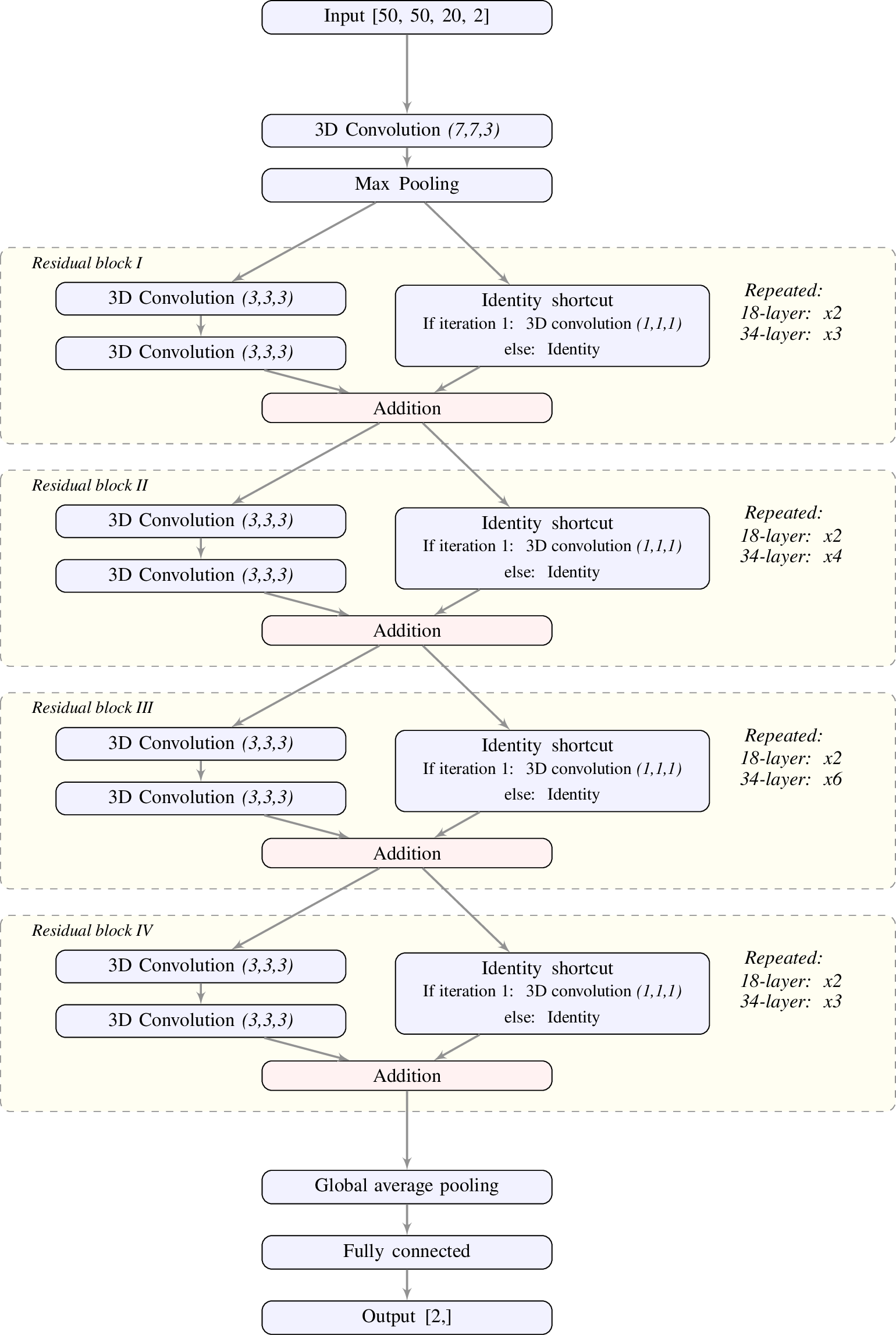

Supplement: S3 Fig — 3D adaptation of the 2D networks from [38] which are available for use in the software. (TIF) [file pcbi.1009074.s003.tif]

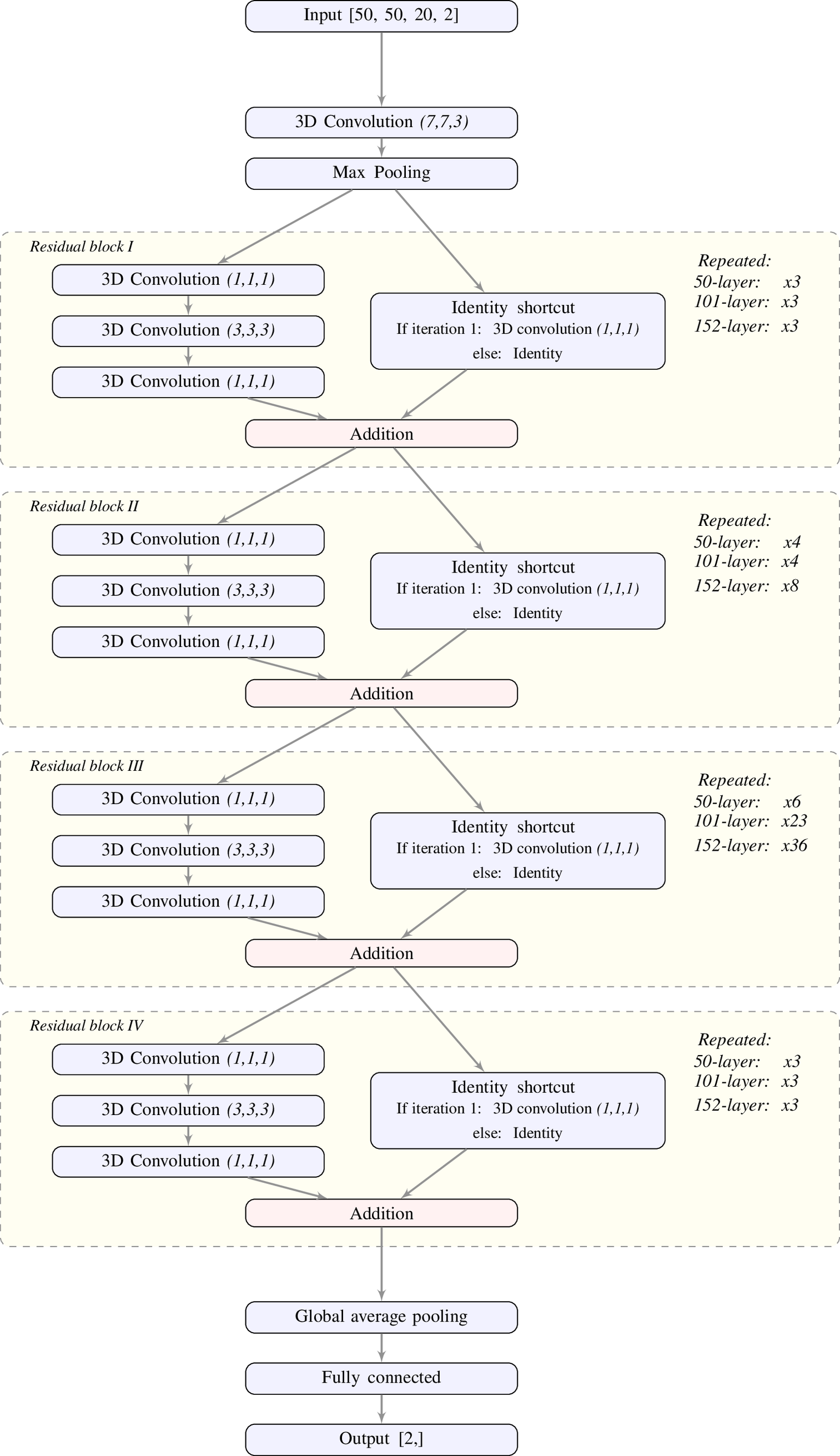

Supplement: S4 Fig — 3D adaptation of the bottleneck 2D networks from [38] which are available for use in the software. The 50-layer bottleneck network is used throughout this study, and is used for the pre-trained model supplied with the software. (TIF) [file pcbi.1009074.s004.tif]
